# NFATc3 mediates the sensitivity of gastric cancer cells to arsenic sulfide

**DOI:** 10.18632/oncotarget.17175

**Published:** 2017-04-18

**Authors:** Xiuli Zhang, Ting Kang, Lian Zhang, Yingying Tong, Wenping Ding, Siyu Chen

**Affiliations:** ^1^ Department of Oncology, Xin Hua Hospital Affiliated to Shanghai Jiao Tong University School of Medicine, Shanghai, China

**Keywords:** As_4_S_4_, NFATc3, c-Myc, sensitivity, gastric cancer

## Abstract

Arsenic sulfide (As_4_S_4_) is the main component of Realgar which is widely used in traditional Chinese medicine. Previously we showed that As_4_S_4_ inhibited the proliferation of colon cancer cells through regulating nuclear factor of activated T cells (NFAT) pathway. Here we explore the role of NFAT in gastric cancer. We showed that As_4_S_4_ inhibited the expression of NFATc1, NFATc3, and NFATc4, and modulated the expression of NFATc2 accompanying with p53. The baseline expression of NFATc3 varied distinctly in gastric cancer cell lines (AGS, MGC803, MKN28, MKN45, and SGC7901) and the sensitivity of these cells to As_4_S_4_ was dissimilar, with AGS and MGC803 cells showing higher sensitivity while the SGC7901 cells relatively resistant. Interestingly, the sensitivity to As_4_S_4_ was correlated with the level of expression of NFATc3, and the cells relatively sensitivity just showing higher expression of NFATc3. Furthermore, NFATc3 expression was significantly higher in gastric cancer tissues compared with the adjacent normal tissues. Our data also showed that, NFATc3 promoted the proliferation of gastric cancer cells by regulating c-Myc. In conclusion, As_4_S_4_ inhibited the proliferation of gastric cancer cells through NFATc3/c-Myc pathway and the diverse sensitivity among different cell lines correlated with the expression level of NFATc3 indicating that NFATc3 may be a potential therapeutic target in gastric cancer.

## INTRODUCTION

Gastric cancer (GC) is an important malignant disease associated with very poor prognosis and the second leading cause of cancer-associated death worldwide, and almost half of all GC cases occur in Asia [1]. Presently, surgical resection and adjuvant or neoadjuvant chemotherapy and radiation remain the principle treatment options of early stage GC [2]. However, the mainstay therapy for the advanced stage GC is systemic chemotherapy, with the 5-year overall survival less than 20% [3]. Recent studies indicate that aberrant expression of a number of genes is associated with gastric carcinogenesis and cancer progression. For example, neogenin-1 promotes gastric cancer cell proliferation and motility, and an IL-17 polymorphism might be the main risk factor for gastric cancer in China and Japan [4, 5]. Therefore, a more thorough understanding of the molecular mechanisms underlying GC development and progression can help improve the treatment and outcome of patients with GC. Nuclear factor of activated T cells (NFAT) was first identified as a transcription factor binding to the interleukin-2 promoter in human T cells [6, 7]. Since then, various NFAT isoforms including NFATc1, NFATc2, NFATc3, NFATc4, and NFAT5 have been identified and characterized. Expression of NFATs is not restricted to T cells, with other cells and tissues—including muscle, bone, neurons, viscera, and skin—all producing various NFATs [8–11]. The biological functions of NFATs include angiogenesis, cardiovascular development, immune regulation, and bone homeostasis. Additionally, recent studies suggest roles for NFATs in the initiation and progression of cancer, with cancer cell proliferation, invasion, metastasis, drug resistance, and the tumor microenvironment all influenced by NFATs [12–14]. For example, NFATc1 promotes proliferation of hepatocellular carcinoma cells [15], NFATc2 increases the invasiveness of breast cancer cells [16], and NFATc3 and NFATc4 promote the progression of colon cancer [17]. Contrastingly, other studies have shown that NFATc2 and NFATc3 can act as tumor suppressors [18, 19]. These findings indicate that the precise functions of different NFATs in cancer are context-dependent.

Our previous study has shown that arsenic sulfide (As_4_S_4_) can regulate NFAT gene expression via the promyelocytic leukemia (PML) and p53 pathways in solid tumor cells [17]. Our current study describes a novel role for NFATc3 in GC. We found that As_4_S_4_ could inhibit GC cell proliferation, and that the sensitivity of these cells to As_4_S_4_ was significantly different. Consistent with our previous studies, As_4_S_4_ modulated the expression of NFATs [17], while the baseline expression of NFATc3 was diverse among cell lines. Furthermore, NFATc3 levels were higher in GC tissues compared with corresponding adjacent normal tissues, and NFATc3 suppressed proliferation of GC cells by regulating c-Myc. Importantly, cells with higher NFATc3 expression levels were more sensitive to As_4_S_4_, while cells with lower NFATc3 expression levels were relatively resistant to As_4_S_4_. Moreover, the sensitivity of GC cells to As_4_S_4_ changed when the expression level of NFATc3 was altered. These findings suggest that expression levels of NFATc3 influence the sensitivity of GC cells to As_4_S_4_, suggesting a potential role for As_4_S_4_ as an anti-cancer therapeutic in appropriate patients.

## RESULTS

### IC_50_ values of As_4_S_4_ vary among GC cell lines

**Figure 1:**
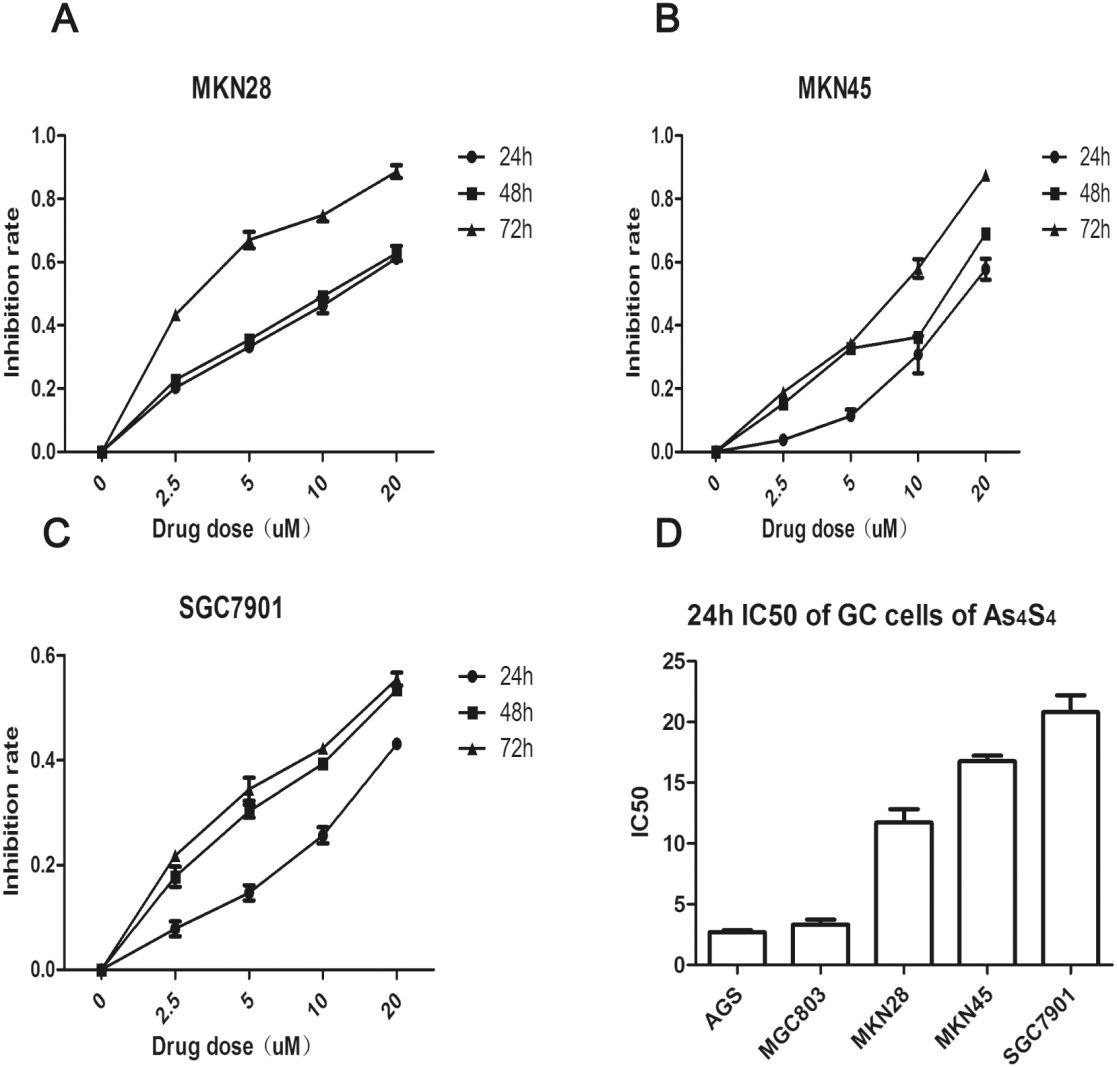
Curves of cell inhibition rates in gastric cancer cells treated with As_4_S_4_ **(A-C)** Dose-and time-dependent curves of cell inhibition rates in cells treated with As_4_S_4_, **(A-C)** represent MKN28, MKN45, SGC7901, respectively. **(D)** The comparisons of the IC_50_ to As_4_S_4_ of five gastric cancer cells. Data represent the mean ± standard deviation of three independent experiments.

We previously reported that AGS and MGC803 have different sensitivities to As_4_S_4_, and that both cell lines were more sensitive than the human gastric epithelium cell line GES-1. The 24 h IC_50_ values of As_4_S_4_ for AGS and MGC803 cells were 2.69 and 3.26 μM, respectively [20]. Therefore, here we treated additional GC cell lines—MKN28, MKN45, and SGC7901—with increasing concentrations of As_4_S_4_ (0, 2.5, 5, 10, and 20 μM) for 24, 48, or 72 h. As_4_S_4_ inhibited the proliferation of all GC cells in a time- and dose-dependent manner. Additionally, the 24 h IC_50_ values for MKN28, MKN45, and SGC7901 cells were 11.71, 16.77, and 20.8 μM, respectively (Figure 1A–1C). Figure 1D demonstrates that the 24 h IC_50_ values of As_4_S_4_ for GC cells varied by almost ten-fold, and that AGS and MGC803 cells were relatively sensitive, while SGC7901 cells were relatively resistant.

### NFAT protein levels in GC cells are modulated by As_4_S_4_

**Figure 2:**
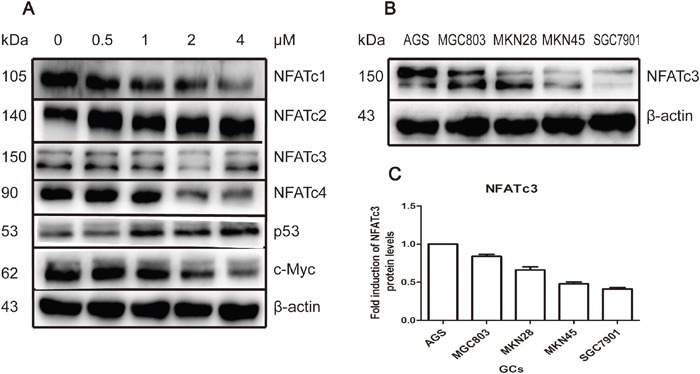
As_4_S_4_ modulated the NFAT protein level; the baseline expression of NFATc3 among GC cell-lines **(A)** AGS cells were treated with As_4_S_4_ in different concentration for 24 hours and NFATc1, NFATc3, NFATc4 and c-Myc were down-regulated while NFATc2 and p53 were up-regulated. **(B)** The baseline expression of protein NFATc3 in AGS, MGC803, MKN28, MKN45 and SGC7901 cells was various distinctly. **(C)** Protein quantification of the Western blot results shown in **(B)**, NFATc3 protein levels were normalized to the β-actin levels and are shown as fold changes relative to the levels of AGS which is as control strain.

We have previously revealed that NFAT proteins are targets of As_4_S_4_ in multiple types of cancer cells including GC cells [17]. Figure 2A shows that treatment of AGS cells with As_4_S_4_ inhibited the expression of NFATc1, NFATc3, and NFATc4, and increased that of NFATc2. Meanwhile, c-Myc was down-regulated and p53 up-regulated. We previously identified similar results in MGC803 cells, except that differential modulation of NFATc2 was identified. This is likely because AGS cells express intact p53, while MGC803 cells have mutated p53 [17].

### Baseline expression of NFATc3 varies dramatically among GC cell lines

We next examined the role of specific members of NFAT family in GC. We first measured the baseline expression levels of NFAT the family members NFATc1, NFATc2, NFATc3, and NFATc4. Western blotting revealed that NFATc3 was expressed at much higher levels in AGS and MGC803 cells compared with SGC7901 cells (Figure 2B). Therefore, GC cells relatively sensitive to As_4_S_4_ (AGS and MGC803) had higher expression levels of NFATc3, while relatively resistant SGC7901 cells had lower levels of NFATc3. However, levels of other NFAT proteins did not exhibit a relationship with cells sensitivity to As_4_S_4_ (Supplementary Figure 1).

### NFATc3 expression levels are higher in GC tissue compared with adjacent normal tissue

**Figure 3:**
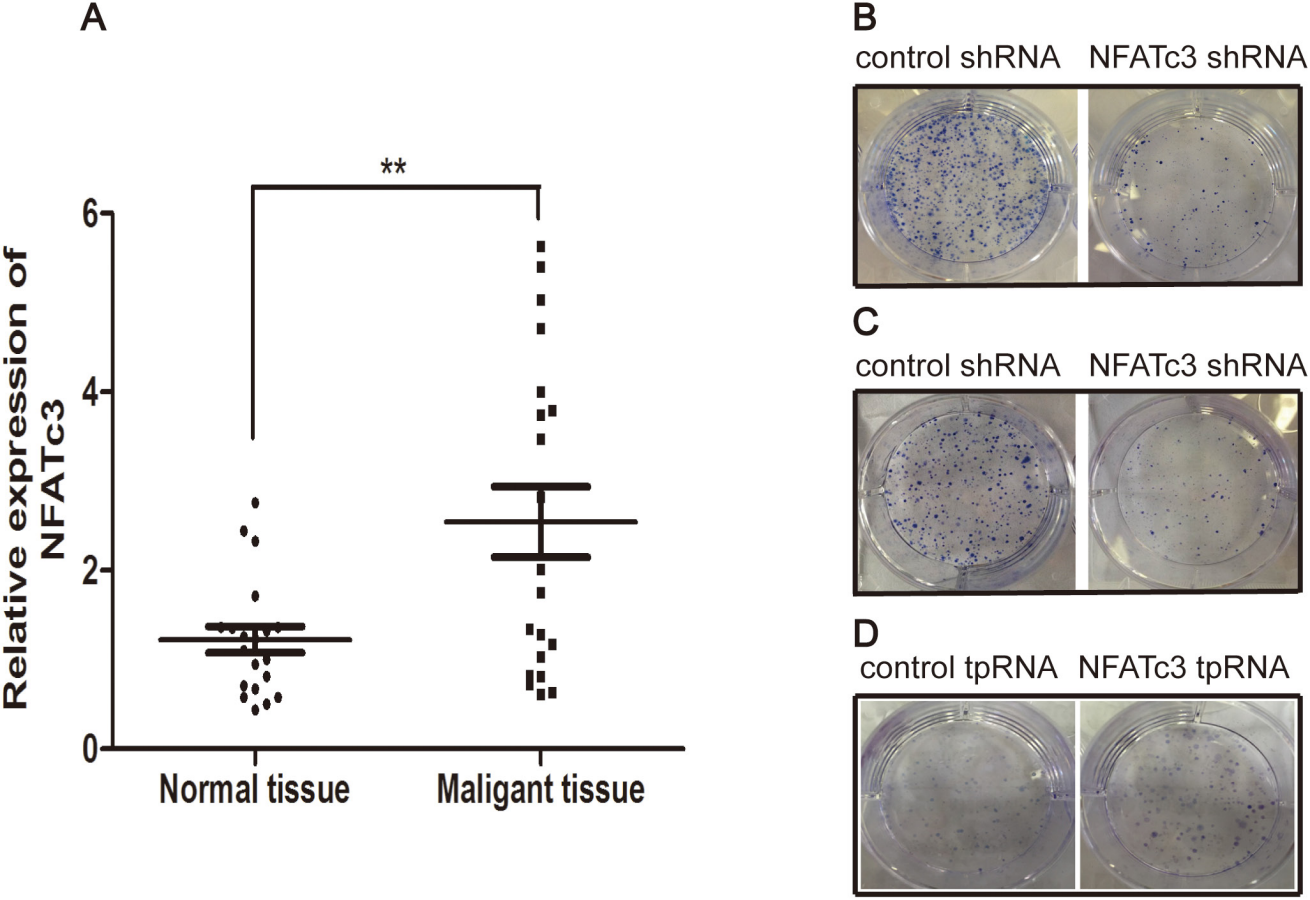
The effect of NFATc3 in GC **(A)** Comparative analysis of NFATc3 expression was determined in 20 pairs of tumor tissues and the corresponding adjacent normal tissues by real time quantitative PCR (qRT-PCR). **P < 0.01 compared to normal tissues. **(B-D)** Giemsa staining of colonies after the transfection. (**B** and **C**) represent AGS, MGC803 respectively, **(D)** represents SGC7901. It shows that NFATc3 could promote colony formation and indicates that it plays a pro-proliferation role in GC cells.

We next evaluated NFATc3 expression in 20 cases of GC samples and the corresponding adjacent normal tissues by real time quantitative PCR. Significantly higher levels of NFATc3 were detected in GC samples than in corresponding non-tumor tissues (Figure 3A, *P* < 0.01). These findings suggest that NFATc3 may serve as an oncogene in GC and the expression of NFATc3 may play an important role in the pathogenesis and development of GC.

### NFATc3 can promote GC cell proliferation *in vitro*

We investigated the function of NFATc3 in GC tumorigenesis by infecting AGS and MGC803 cells with lentiviruses carrying NFATc3 shRNA, and transfecting SGC7901 cells with plasmid expressing NFATc3 tpRNA. Figure 3B–3D reveals that knockdown of NFATc3 in AGS and MGC803 cells significantly inhibited colony formation, while over-expression of NFATc3 in SGC7901 cells promoted colony formation. Taken together, these findings indicate that NFATc3 promotes the proliferation of GC cells.

### NFATc3 modulates other members of the NFAT family and may promote GC cell proliferation through c-Myc

**Figure 4:**
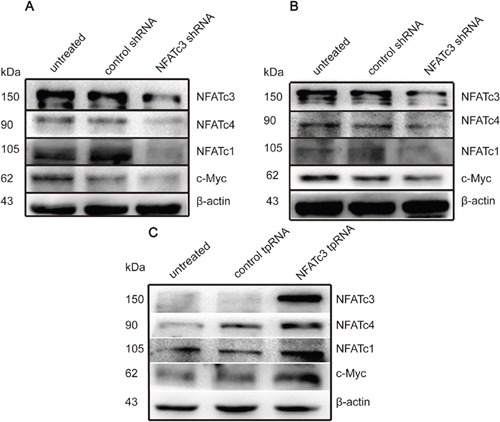
NFATc3 regulated the expression of c-Myc and other members of NFAT family **(A, B)** Western blotting shows the expression of c-Myc, NFATc1 and NFATc4 can be suppressed when the cells transfected with Lentiviruses carrying NFATc3 shRNA. **(C)** Western blotting shows the expression of c-Myc, NFATc1 and NFATc4 can be up-regulated when the cells transfected with plasmid carrying NFATc3 tpRNA. **(A-C)** represent AGS, MGC803 and SGC7901cells, respectively.

We next investigated the mechanisms of how NFATc3 regulates GC cells by measuring expression levels of other NFAT family members after knockdown or over-expression of NFATc3. Knockdown of NFATc3 in AGS and MGC803 cells downregulated NFATc1, NFATc4, and the oncogene c-Myc (Figure 4A, 4B). Meanwhile, over-expressing NFATc3 in SGC7901 cells up-regulated NFATc1, NFATc4, and c-Myc (Figure 4C). These findings reveal interactions between NFAT family members, and suggest that NFATc3 may promote the proliferation of GCs *in vitro* by using c-Myc as one of its target genes.

### The sensitivity of GC cells to As_4_S_4_ is associated with the baseline expression of NFATc3

**Figure 5:**
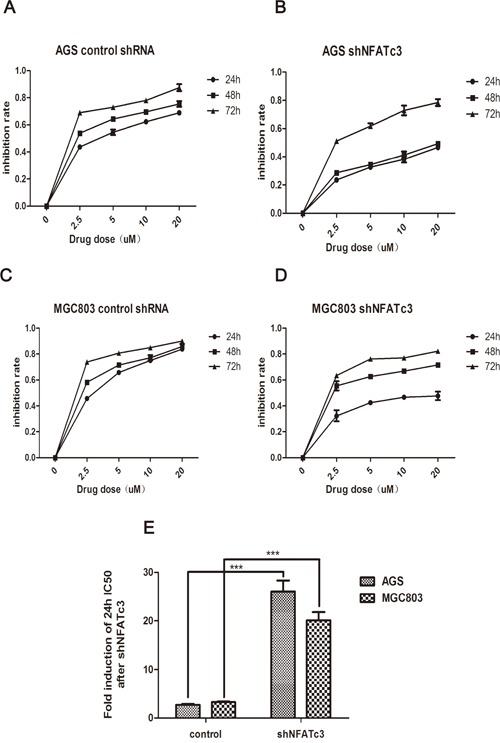
Curves of cell inhibition rates in GC cells treated with As_4_S_4_ after the transfection of the Lentiviruses carrying NFATc3 shRNA **(A, C)** Dose- and time-dependent curves of cell inhibition rates in cells treated with As_4_S_4_ after the transfection of the Lentiviruses carrying control shRNA. **(B, D)** Dose- and time-dependent curves of cell inhibition rates in cells treated with As_4_S_4_ after the transfection of the Lentiviruses carrying NFATc3 shRNA. **(A, B)** represent AGS cell, **(C, D)** represent MGC803 cell. **(E)** The fold change of 24h IC_50_ of GC cells to As_4_S_4_ after the transfection of the Lentiviruses carrying NFATc3 shRNA. ***P < 0.001.

**Figure 6:**
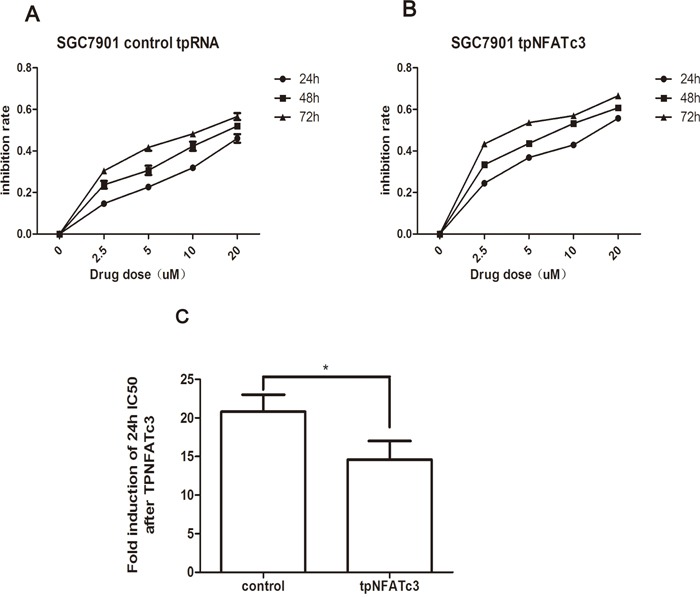
Curves of cell inhibition rates in SGC7901 cell treated with As_4_S_4_ after the transfection of the plasmid carrying NFATc3 tpRNA **(A)** Dose- and time-dependent curves of cell inhibition rates in cells treated with As_4_S_4_ after the transfection of the plasmid carrying control tpRNA. **(B)** Dose- and time-dependent curves of cell inhibition rates in cells treated with As_4_S_4_ after the transfection of the plasmid carrying NFATc3 tpRNA. **(C)** The fold change of 24h IC_50_ of SGC7901 cells to As_4_S_4_ after the transfection of the plasmid carrying NFATc3 tpRNA. *P < 0.05.

Finally, we examined whether the IC_50_ values for As_4_S_4_ were altered by changes to NFATc3 levels. Figure 5 shows that the 24-h IC_50_ values of As_4_S_4_ for AGS and MGC803 cells in which NFATc3 had been silenced were 26.06 and 20.12 μM, respectively. These findings reflect decreases in the sensitivity of AGS and MGC803 cells to As_4_S_4_ of 9.69- and 6.17-fold, respectively. Meanwhile, over-expression of NFATc3 in SGC7901 cells decreased the 24-h IC_50_ of As_4_S_4_ from 20.8 to 14.59 μM (Figure 6). These results reveal that the GC cells with higher expression levels of NFATc3 are more sensitive to As_4_S_4_.

## DISCUSSION

GC is an aggressive neoplasm with high prevalence, poor prognosis, and limited treatment options [21]. Furthermore, half of cases worldwide occur in China. Presently, the efficacy of chemotherapeutic drugs in clinical use for the treatment of advanced-stage GC is unsatisfactory. Therefore, there is an urgent need to identify the key factors involved in the disease progression and new effective targets for drug development. Our previous study demonstrated that As_4_S_4_ can exert antitumor effects in both gastric and colon cancers, and that As_4_S_4_ regulates the expression of NFAT via PML and p53. Furthermore, we revealed that NFATc3 can promote tumorigenesis by regulating c-Myc [17].

The NFAT family has important roles in modulating biological behavior of malignant tumors [12]. For example, the functions of NFAT family members have been elaborated in pancreatic cancer [22–24], leukemia [25, 26], breast cancer [27, 28], and melanoma [29].

However, there are few reports concerning the role of NFAT family members in GC. In addition to their roles in influencing cell proliferation, recent studies have identified important roles for NFAT in modulating drug resistance, NFATc1 over-expression renders pancreatic cancer cells less responsive to treatment with phospho-sulindac [30]. Furthermore, impairment of NFAT activity facilitates leukemia cell elimination by the BCR-ABL inhibitor dasatinib, and NFAT inhibition augments the anti-cancer effects of vemurafenib and trametinib in melanoma [31, 32].

Additionally, inhibition of the calcineurin–NFAT pathway by cyclosporine A reverses resistance to ABT-737 in activated T lymphocytes. Meanwhile, others have found that combination treatment of NFAT inhibition with cyclosporine A could reverse resistance to the MEK inhibitor selumetinib in a patient-derived tumor xenograft model of colorectal cancer [33, 34]. However, specific roles for other members of the NFAT family in mediating resistance to anti-cancer drugs have not been described.

Our previous studies indicated that different cancer cells exhibited distinct sensitivities to As_4_S_4_. Therefore, we collected a series of gastric (AGS, MGC803, MKN28, MKN45, and SGC7901) and colon (HCT116, SW480, HT-29, LoVo, RKO, and Caco-2) cancer cell lines to explore their sensitivity to As_4_S_4_. As_4_S_4_ inhibited the proliferation of all cell lines, and the range of IC_50_ values among GC cells is wider than that for colon cancer cells. We previously found that As_4_S_4_ downregulated NFATc1, NFATc3, and NFATc4 levels in colon cancer cells, while—as long as p53 was intact—NFATc2 was up-regulated [17]. Similar results were found for GC MGC803 cells, except that NFATc2 was down-regulated because p53 is mutated in these cells [17]. We have further explored the role of NFATs in GC by detecting the baseline expression levels of NFAT family members including NFATc1–c4 in the aforementioned GC cell lines. Interestingly, the expression of NFATc3—but not other NFAT family members—varied among GC cells. However, this phenomenon was not obvious in the colon cancer cell lines examined. We then investigated whether NFATc3 expression is altered in GC patients by collecting 20 pairs of GC and corresponding adjacent normal tissues and measuring NFATc3 levels. NFATc3 was over-expressed in GC tissue compared with adjacent non-tumor tissue, consistent with our previous study of colon cancer patient samples [17], indicating that NFATc3 may act as a pro-tumorigenesis factor.

Consistently, altering the expression of NFATc3 revealed that it promotes the proliferation of GC cells and modulates the expression of c-Myc. In our previous research by chromatin IP (CHIP), we confirmed that NFATc3 indeed bond to the c-Myc promoter in colon cancer to yield increased levels of c-Myc [17]. c-Myc influences myriad cellular functions, including cell proliferation, survival, and microRNA expression [35]. Taken together with our previous findings that As_4_S_4_ inhibits the proliferation of GC [20], we hypothesize that As_4_S_4_ inhibits GC cell proliferation through down-regulation of the NFATc3/c-Myc pathway.

We have revealed that NFATc3 promotes the proliferation of GC cells. Furthermore, NFATc3 expression is associated with the IC_50_ value for As_4_S_4_, with higher levels of NFATc3 conferring increased sensitivity to As_4_S_4_, and vice versa. Consistently, knockdown of NFATc3 in cells relatively sensitive to As_4_S_4_ (AGS and MGC803) decreased their sensitivity. Meanwhile, over-expression of NFATc3 in relatively resistant cells (SGC7901) increased their sensitivity to As_4_S_4_. These findings support the notion that the sensitivity of GC cell lines to As_4_S_4_ is associated with NFATc3 expression levels.

NFATc3 also regulates the expression of other NFAT family members. Knockdown of NFATc3 suppressed the expression of NFATc1 and NFATc4, while over-expression of NFATc3 had opposite effects. Our findings further highlight the versatility of the NFAT transcription factor family in modulating the behavior of cancer cells.

Taken together, our results show that the baseline expression of NFATc3 is related to the sensitivity to As_4_S_4_. However, these interesting results are all based on *in vitro* studies, still need to be further validated *in vivo*. Now that NAFTc3 could mediate the sensitivity of GC cells to As_4_S_4_, whether it is associated with sensitivity of other drugs in other cancers should also be explored.

Collectively, the results obtained in the present study have demonstrated a novel role for NFATc3 in modulating drug response in GC. Importantly, these findings may implicate a new potential therapeutic target for the treatment of this deadly malignancy.

## MATERIALS AND METHODS

### Ethical statement

Investigation has been conducted in accordance with the ethical standards and according to the Declaration of Helsinki and according to national and international guidelines and has been approved by the authors' institutional review board.

### Cell culture and reagents

The human GC cell lines AGS, MGC803, and SGC7901 were obtained from the Cell Bank, Chinese Academy of Sciences (Shanghai, People's Republic of China), MKN28 and MKN45 cells were obtained from Institute of Digestive of Shanghai Ruijin Hospital affiliated with Shanghai Jiao Tong University. No ethics statement was required from the institutional review board for the use of these cell lines. AGS cells were cultured in Dulbecco's modified Eagle medium (DMEM)/F12 1:1 medium (Hyclone, Logan, UT, USA). MGC803 and SGC7901cells were cultured in RPMI 1640 medium (Gibco, Waltham, MA, USA). MKN28 and MKN45 cells were cultured in DMEM medium (Hyclone, Logan, UT, USA). All medium was supplemented with 10% fetal bovine serum (Gibco, Waltham, MA, USA). All cells were cultured at 37°C in an atmosphere of 95% air and 5% CO_2_. Highly purified As_4_S_4_ was supplied by the Shanghai Institute of Hematology (Shanghai, People's Republic of China) and was prepared as previously described [20]. The stock solution of As_4_S_4_ (133.36 μM) was diluted in complete culture medium to obtain working solutions. 3-(4, 5-dimethylthiazol-2-yl)-2, 5-diphenyltetrazolium bromide (MTT) was purchased from Sigma-Aldrich (St Louis, MO, USA). Antibodies for NFATAc1, NFATAc3, and NFATAc4 were purchased from Sigma-Aldrich (St Louis, MO, USA), Santa Cruz Biotechnology (Santa Cruz, CA, USA), and Abcam (Cambridge, MA, USA), respectively. Antibody for c-Myc was from Cell Signaling Technology (Beverly, MA, USA), while anti-β-actin antibody was obtained from Proteintech Group (Wuhan, Hubei, People's Republic of China).

### Cytotoxicity assay

The cytotoxicity assay was performed as previously described [36]. Briefly, MKN28, MKN45, or SGC7901 cells (5 × 10^3^ cells/well, in 90 μL medium) were seeded into 96-well plates and allowed to attach. The following day, As_4_S_4_ at different concentrations (in 10 μL volumes) was administered to cells. Then, 10 μL MTT (5 mg/mL) was added to designated wells after 24, 48, or 72 h and incubated at 37°C for 4 h. Thereafter, 150 μL sodium dodecyl sulfate (SDS) was added to each well and incubated at 37°C overnight. The optical density at 562 nm was measured using a microplate reader (Bio-TEK, Vermont, USA). Cells incubated without any treatment were used as control. Culture medium without cells was used as blank control. The IC_50_ (concentration causing 50% inhibition of cell growth compared with the control) value of As_4_S_4_ for each of the tumor cell lines was calculated after 24 h. Data were analyzed based on three independent experiments. The IC_50_ values for AGS and MGC803 cells were calculated using the same method described in our previous study [20].

### Western blotting

Total proteins were extracted using enhanced radioimmunoprecipitation assay lysis buffer (Beyotime, Shanghai, China) containing 1% phenylmethanesulfonyl fluoride (Beyotime) on ice. Protein concentrations were determined using a microplate reader (Bio-TEK) with the enhanced bicinchoninic acid protein assay kit (Beyotime). Equal amounts of protein in each lane were separated by different SDS-polyacrylamide gel electrophoresis according to the molecular weight of measured proteins and transferred onto a polyvinylidene difluoride membrane (Millipore, Billerica, MA, USA). After blocking the membrane in 5% non-fat milk, the membrane was then incubated with diluted primary antibodies at 4°C overnight. Membranes were then washed three times with tris-buffered saline with Tween 20 (the percentage of Tween 20 was 0.05%) and incubated with labeled secondary antibody at room temperature for 1 h, and then washed thrice again. Finally, proteins were detected using the ECL Plus kit (Millipore), and images were acquired using the GelDoc XR System (Bio-rad, USA).

### Infection with shRNA-encoding lentivirus

Lentiviruses (pHBLV-U6-Zsgreen) encoding NFATc3 shRNA (shNFATc3) were provided by HanBio (Shanghai, China). AGS and MGC803 cells were grown to 30–40% confluence in 6-well culture plates and then infected with lentivirus encoding shRNA according to the manufacturer's instructions. Briefly, 5 μL Polybrene was added to each well and incubated at 37°C for 30 min, followed by addition of required amounts lentivirus according to the multiplicity of infection of specific cells. Infection efficiency was observed by fluorescence microscopy on the basis of GFP expression, and further confirmed by measuring protein levels of NFATc3. After a 12 h incubation at 37°C, cells were washed and incubated with fresh medium for an additional 60 h before analysis; culture medium was replaced daily. The shRNA sequences were the same as those previously used [17].

### pMAX-clover-NFATc3 plasmid construction and transfection

The pMAX-clover-NFATc3 vector for over-expression of NFATc3 was constructed by PCR amplification of the primary NFATc3 sequence (3228 bp) from human cDNA using the primers 5′-AGAATTCATGACTACTGCAAACTGTGG-3′ (forward) and 5′-GCGTCGACTTAGAGCCCATCAGATCTTC-3′ (reverse), and cloning of this sequence downstream of the clover sequence in the pMAX-clover vector using the EcoRI/SalI sites. SGC7901 cells were grown in 6-well culture plates and then transfected with pMAX-clover-NFATc3 using Lipofectamine 2000 (Invitrogen, USA) according to the manufacturer's instructions. Briefly, Lipofectamine 2000 and pMAX-clover-NFATc3 were diluted in opti-MEM medium, incubated for 5 min, mixed together, and then incubated for 30 min at room temperature. Finally, complexes were added to wells containing cells. The transfection efficiency was observed as described above.

### MTT and colony formation assays

AGS and MGC803 cells infected with lentiviruses carrying shNFATc3 and SGC7901 cells transfected with plasmid encoding NFATc3 were subjected to MTT assay. MTT assays were conducted in a similar manner to the cytotoxicity assay described above. For the colony formation assay, cells were trypsinized to generate a single-cell suspension, and 2000 cells/well were seeded into 6-well plates followed by incubation under normal conditions for 2 weeks. Colonies were fixed with 4% paraformaldehyde for 15 min at room temperature and then stained with Giemsa staining fluid for 20 min. Colonies were then counted under a light microscope.

### Patients and tissue samples

The GC samples and their corresponding adjacent normal tissues were obtained from 20 patients diagnosed with GC and treated with surgery at the Xinhua Hospital Affiliated with Shanghai Jiao Tong University of Medicine (Shanghai, China). All samples and clinical information were obtained with informed consent from the patient or their family. Tissues were immediately snap-frozen in liquid nitrogen after surgery and stored at −80°C until RNA extraction. This study was approved by the ethics committee of Xinhua Hospital Affiliated to Shanghai Jiao Tong University.

### Statistical analysis

SPSS software version 20.0 (IBM Corporation, Armonk, NY, USA) was used for all statistical analyses. Experiments were repeated independently three times and all data are presented as the means ± standard deviation (SD). Data analysis was performed using one-way analysis of variance, followed by either the least significant difference procedure (if variance was equal) or the Games–Howell procedure (if variance was unequal). A two-sided *P*-value of < 0.05 was considered statistically significant.

## SUPPLEMENTARY FIGURE



## Abbreviations

As_4_S_4_: arsenic sulfide
NFAT: nuclear factor of activated T cells
GC: gastric cancer
PML: promyelocytic leukemia
