# Supplementary material for: NFATc3 mediates the sensitivity of gastric cancer cells to arsenic sulfide
Source: Oncotarget. 2017 Apr 18;8(32):52735–45. doi: 10.18632/oncotarget.17175 (PMC5581065; doi:10.18632/oncotarget.17175)
Supplement: Supplementary file 1 [file oncotarget-08-52735-s001.pdf]

NFATc3 mediates the sensitivity of gastric cancer cells to arsenic sulfide

SUPPLEMENTARY FIGURE

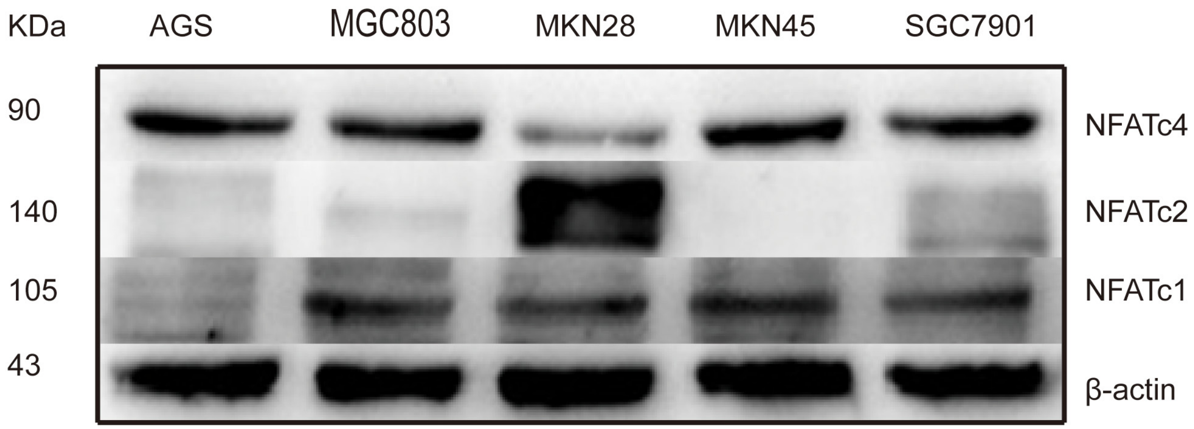

Supplementary Figure 1: The baseline expression of other members of NFAT family including NFATc1, NFATc2 and NFATc14 in AGS, MGC803, MKN28, MKN45 and SGC7901.
